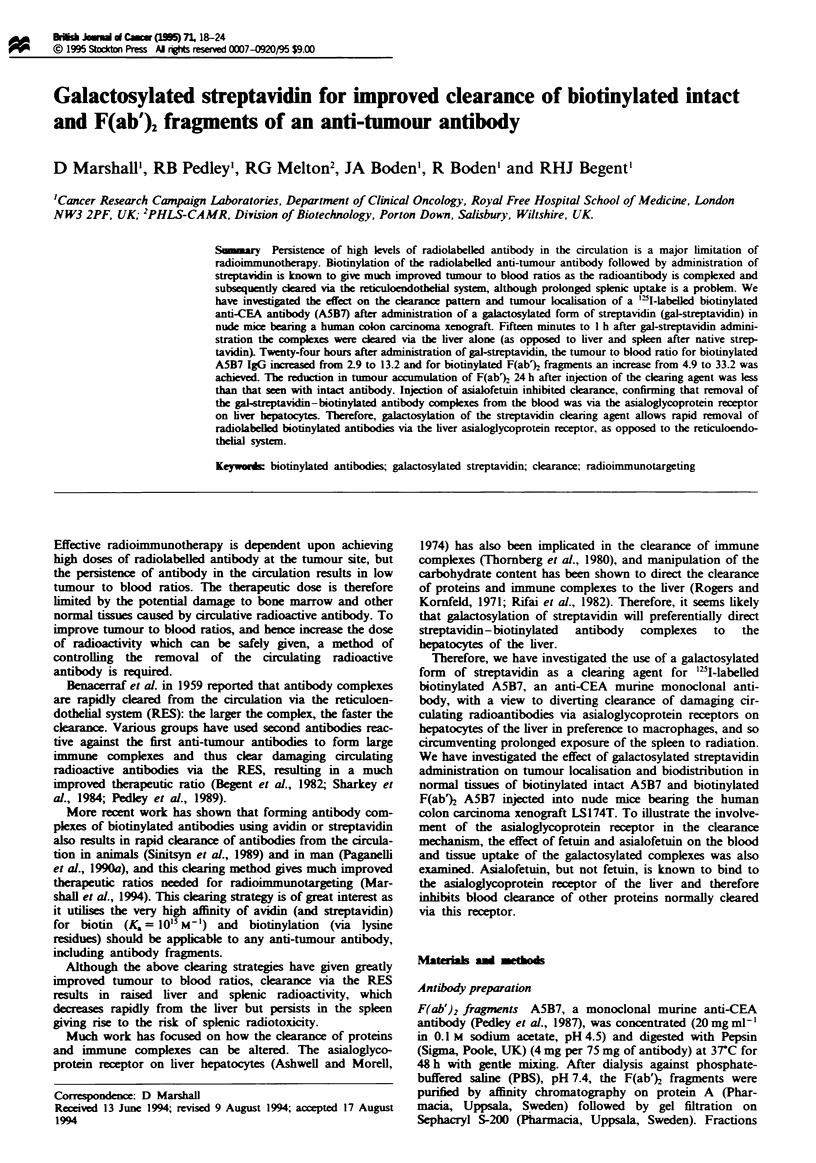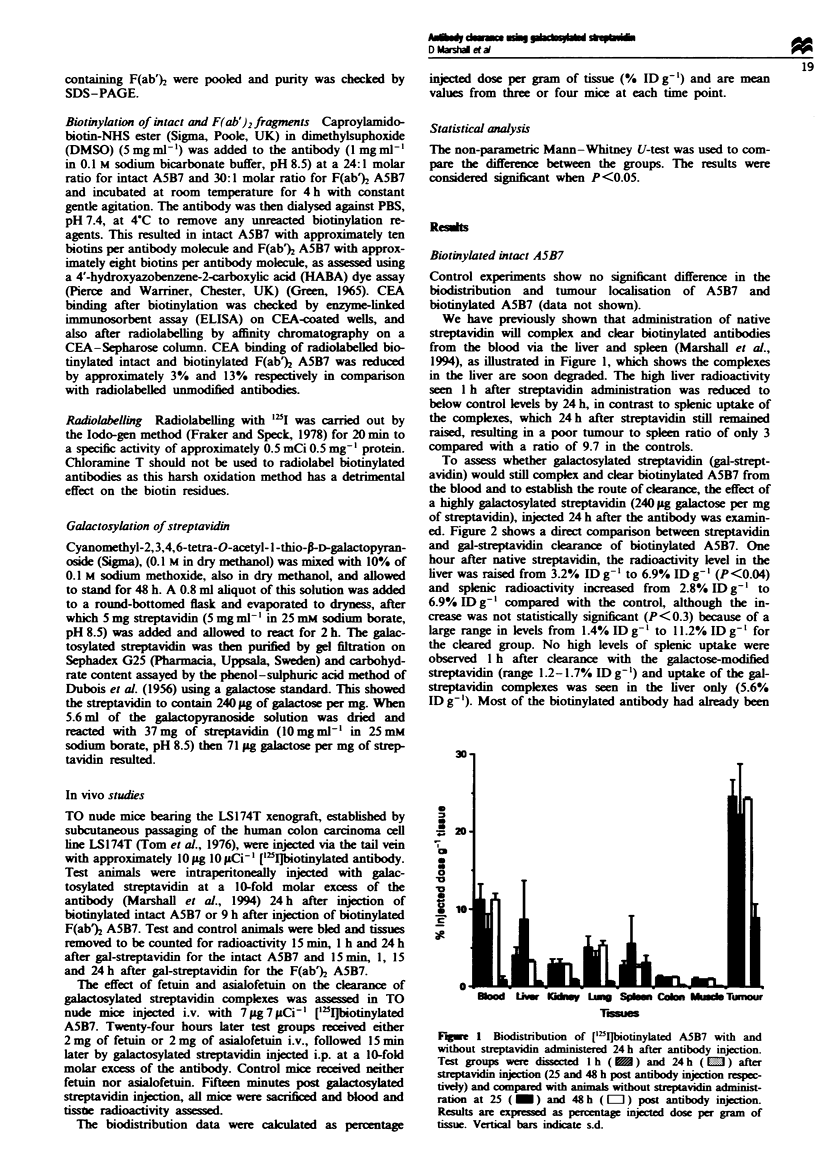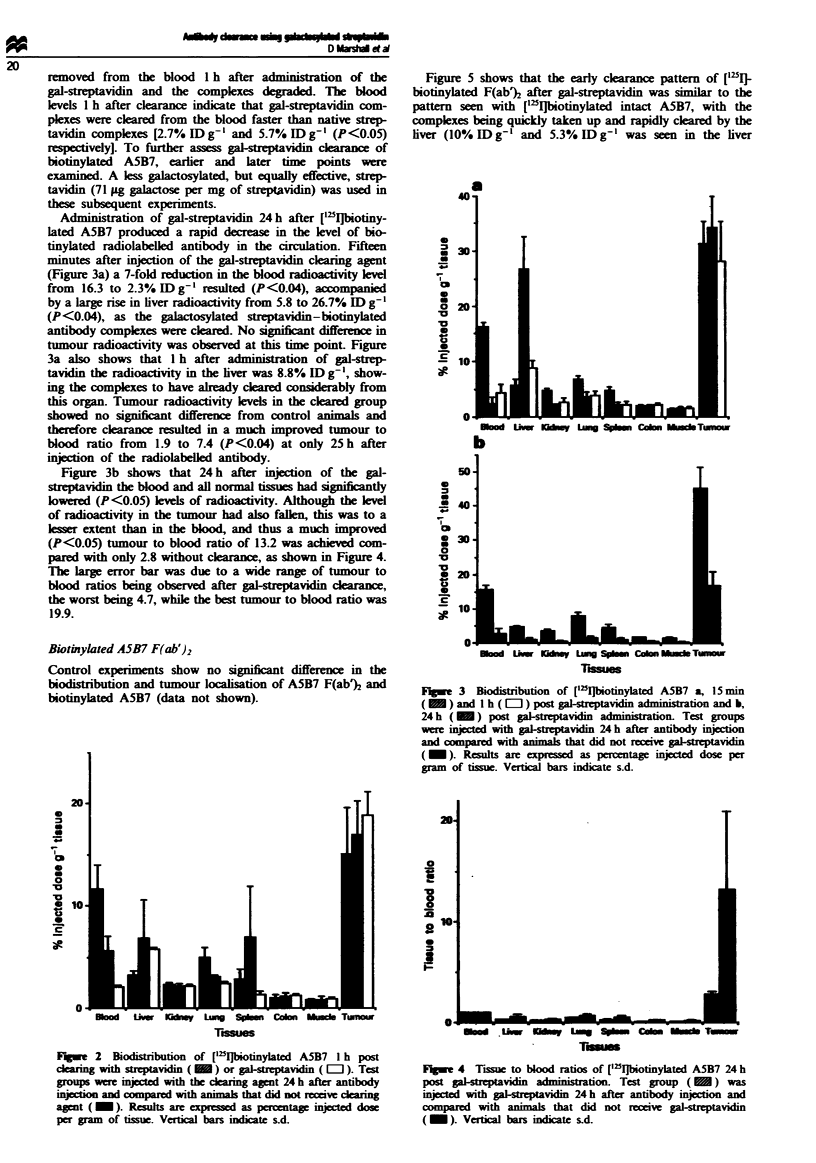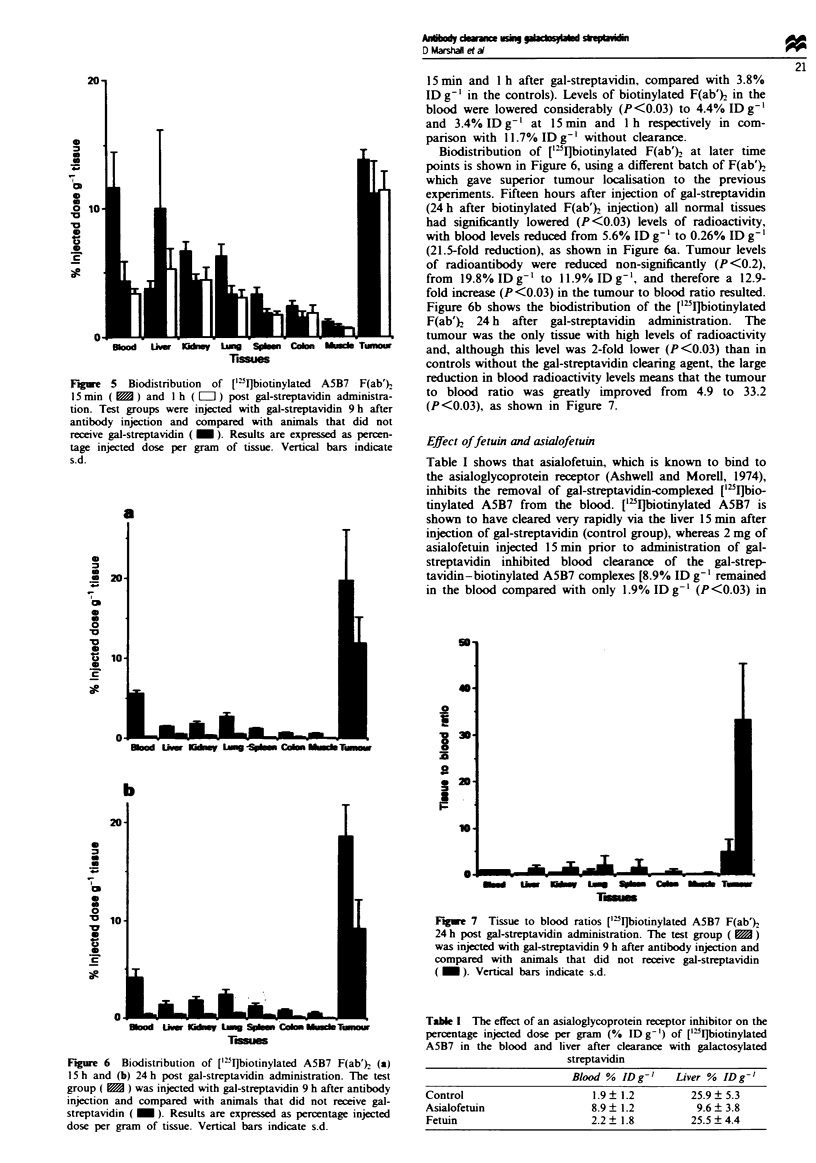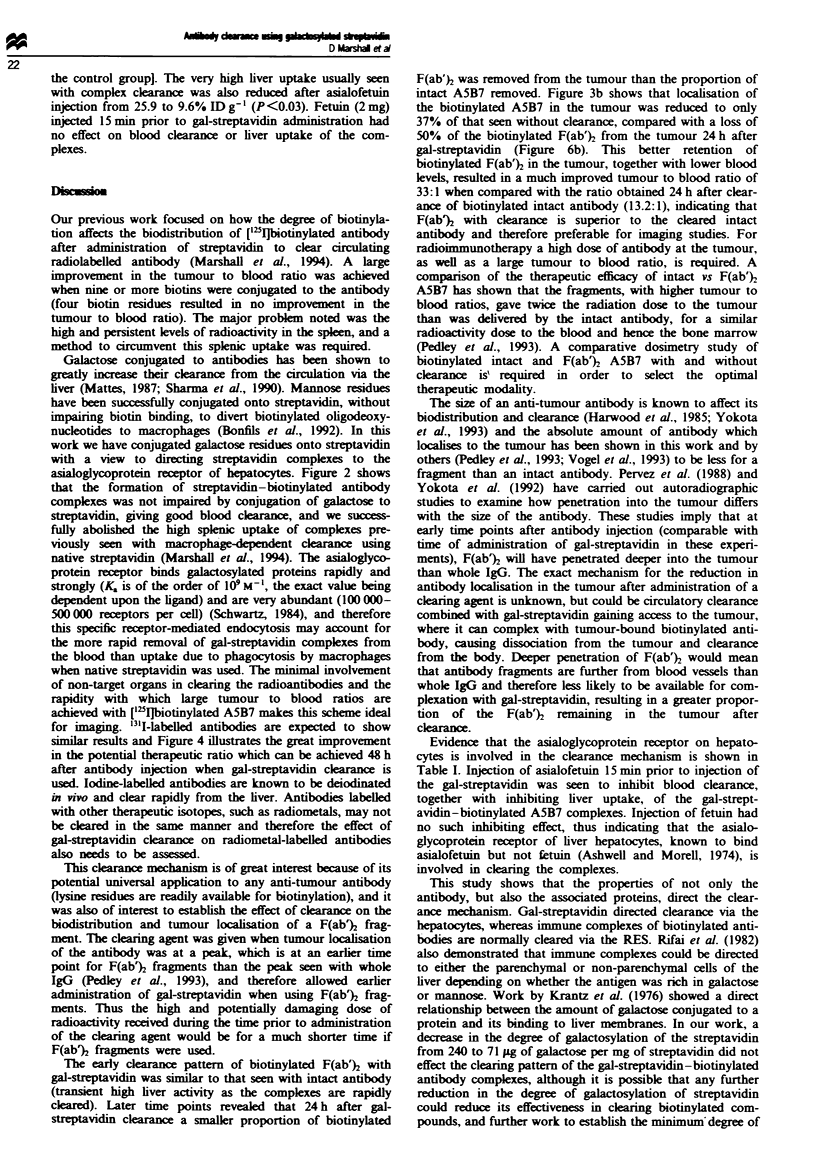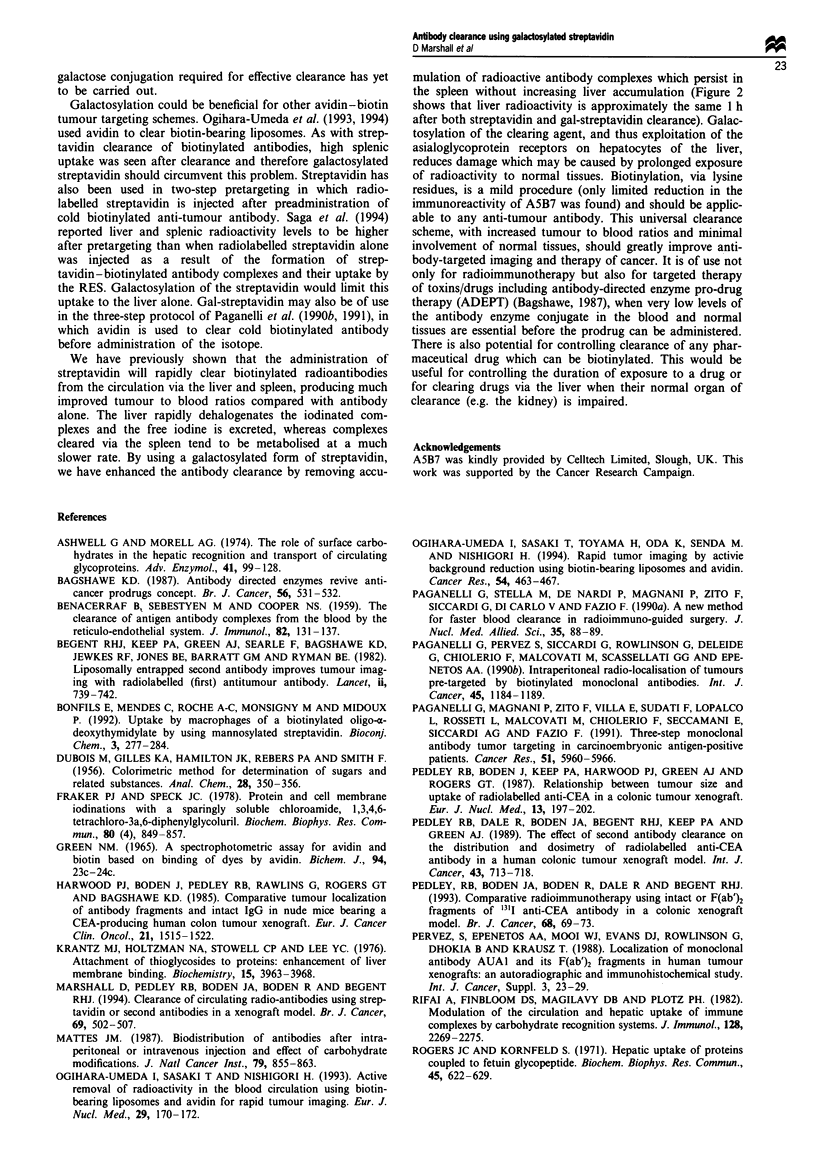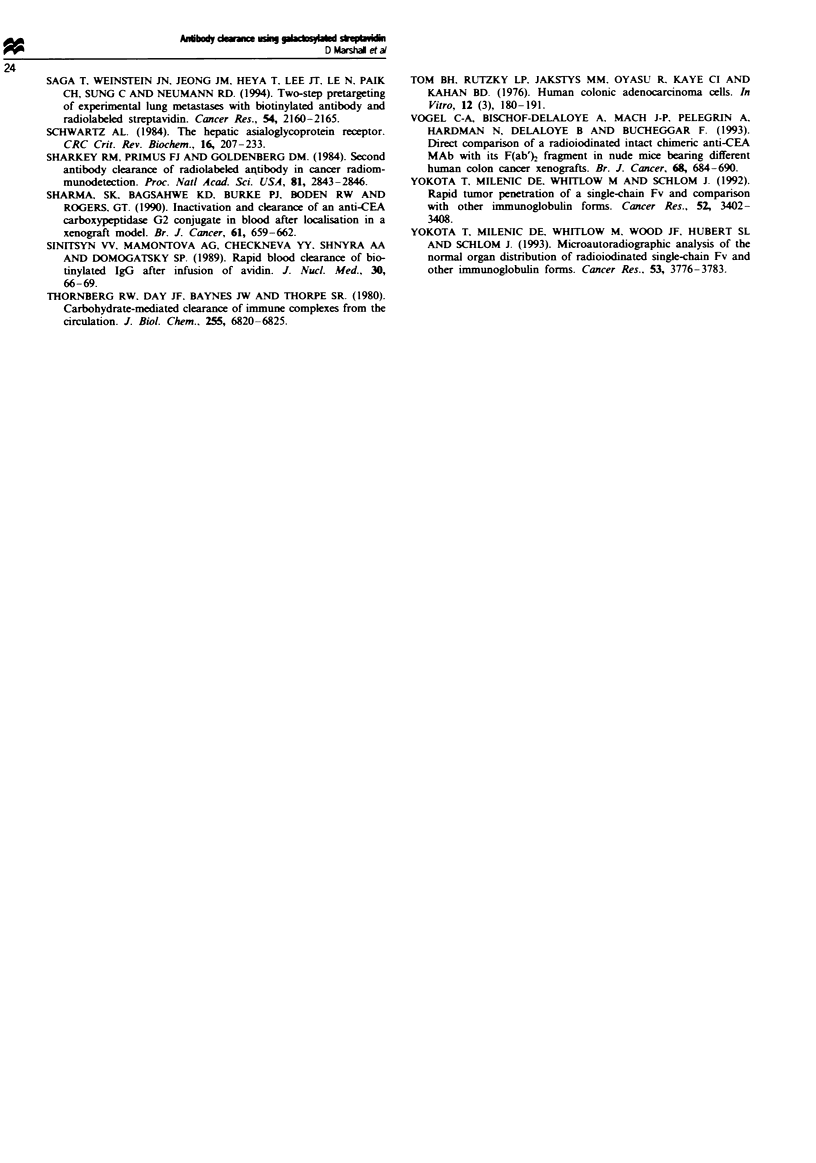# Galactosylated streptavidin for improved clearance of biotinylated intact and F(ab')2 fragments of an anti-tumour antibody.

**DOI:** 10.1038/bjc.1995.5

**Published:** 1995-01

**Authors:** D. Marshall, R. B. Pedley, R. G. Melton, J. A. Boden, R. Boden, R. H. Begent

**Affiliations:** Department of Clinical Oncology, Royal Free Hospital School of Medicine, London, UK.

## Abstract

**Images:**


British Journal of Cancer (1995) 71, 18-24

© 1995 Stockton Press All rights reserved 0007-0920/95 $9.00

### Galactosylated streptavidin for improved clearance of biotinylated intact and F(ab')$_2$ fragments of an anti-tumour antibody

D Marshall[1], RB Pedley[1], RG Melton[2], JA Boden[1], R Boden[1] and RHJ Begent[1]

[1]*Cancer Research Campaign Laboratories, Department of Clinical Oncology, Royal Free Hospital School of Medicine, London NW3 2PF, UK;* [2]*PHLS-CAMR, Division of Biotechnology, Porton Down, Salisbury, Wiltshire, UK.*

**Summary** Persistence of high levels of radiolabelled antibody in the circulation is a major limitation of radioimmunotherapy. Biotinylation of the radiolabelled anti-tumour antibody followed by administration of streptavidin is known to give much improved tumour to blood ratios as the radioantibody is complexed and subsequently cleared via the reticuloendothelial system, although prolonged splenic uptake is a problem. We have investigated the effect on the clearance pattern and tumour localisation of a $^{125}$I-labelled biotinylated anti-CEA antibody (A5B7) after administration of a galactosylated form of streptavidin (gal-streptavidin) in nude mice bearing a human colon carcinoma xenograft. Fifteen minutes to 1 h after gal-streptavidin administration the complexes were cleared via the liver alone (as opposed to liver and spleen after native streptavidin). Twenty-four hours after administration of gal-streptavidin, the tumour to blood ratio for biotinylated A5B7 IgG increased from 2.9 to 13.2 and for biotinylated F(ab')$_2$ fragments an increase from 4.9 to 33.2 was achieved. The reduction in tumour accumulation of F(ab')$_2$ 24 h after injection of the clearing agent was less than that seen with intact antibody. Injection of asialofetuin inhibited clearance, confirming that removal of the gal-streptavidin-biotinylated antibody complexes from the blood was via the asialoglycoprotein receptor on liver hepatocytes. Therefore, galactosylation of the streptavidin clearing agent allows rapid removal of radiolabelled biotinylated antibodies via the liver asialoglycoprotein receptor, as opposed to the reticuloendothelial system.

**Keywords:** biotinylated antibodies; galactosylated streptavidin; clearance; radioimmunotargeting

Effective radioimmunotherapy is dependent upon achieving high doses of radiolabelled antibody at the tumour site, but the persistence of antibody in the circulation results in low tumour to blood ratios. The therapeutic dose is therefore limited by the potential damage to bone marrow and other normal tissues caused by circulative radioactive antibody. To improve tumour to blood ratios, and hence increase the dose of radioactivity which can be safely given, a method of controlling the removal of the circulating radioactive antibody is required.

Benacerraf *et al.* in 1959 reported that antibody complexes are rapidly cleared from the circulation via the reticuloendothelial system (RES): the larger the complex, the faster the clearance. Various groups have used second antibodies reactive against the first anti-tumour antibodies to form large immune complexes and thus clear damaging circulating radioactive antibodies via the RES, resulting in a much improved therapeutic ratio (Begent *et al.*, 1982; Sharkey *et al.*, 1984; Pedley *et al.*, 1989).

More recent work has shown that forming antibody complexes of biotinylated antibodies using avidin or streptavidin also results in rapid clearance of antibodies from the circulation in animals (Sinitsyn *et al.*, 1989) and in man (Paganelli *et al.*, 1990*a*), and this clearing method gives much improved therapeutic ratios needed for radioimmunotargeting (Marshall *et al.*, 1994). This clearing strategy is of great interest as it utilises the very high affinity of avidin (and streptavidin) for biotin ($K_a = 10^{15}\ M^{-1}$) and biotinylation (via lysine residues) should be applicable to any anti-tumour antibody, including antibody fragments.

Although the above clearing strategies have given greatly improved tumour to blood ratios, clearance via the RES results in raised liver and splenic radioactivity, which decreases rapidly from the liver but persists in the spleen giving rise to the risk of splenic radiotoxicity.

Much work has focused on how the clearance of proteins and immune complexes can be altered. The asialoglycoprotein receptor on liver hepatocytes (Ashwell and Morell, 1974) has also been implicated in the clearance of immune complexes (Thornberg *et al.*, 1980), and manipulation of the carbohydrate content has been shown to direct the clearance of proteins and immune complexes to the liver (Rogers and Kornfeld, 1971; Rifai *et al.*, 1982). Therefore, it seems likely that galactosylation of streptavidin will preferentially direct streptavidin-biotinylated antibody complexes to the hepatocytes of the liver.

Therefore, we have investigated the use of a galactosylated form of streptavidin as a clearing agent for $^{125}$I-labelled biotinylated A5B7, an anti-CEA murine monoclonal antibody, with a view to diverting clearance of damaging circulating radioantibodies via asialoglycoprotein receptors on hepatocytes of the liver in preference to macrophages, and so circumventing prolonged exposure of the spleen to radiation. We have investigated the effect of galactosylated streptavidin administration on tumour localisation and biodistribution in normal tissues of biotinylated intact A5B7 and biotinylated F(ab')$_2$ A5B7 injected into nude mice bearing the human colon carcinoma xenograft LS174T. To illustrate the involvement of the asialoglycoprotein receptor in the clearance mechanism, the effect of fetuin and asialofetuin on the blood and tissue uptake of the galactosylated complexes was also examined. Asialofetuin, but not fetuin, is known to bind to the asialoglycoprotein receptor of the liver and therefore inhibits blood clearance of other proteins normally cleared via this receptor.

### Materials and methods

### *Antibody preparation*

*F(ab')$_2$ fragments* A5B7, a monoclonal murine anti-CEA antibody (Pedley *et al.*, 1987), was concentrated (20 mg ml$^{-1}$ in 0.1 M sodium acetate, pH 4.5) and digested with Pepsin (Sigma, Poole, UK) (4 mg per 75 mg of antibody) at 37°C for 48 h with gentle mixing. After dialysis against phosphate-buffered saline (PBS), pH 7.4, the F(ab')$_2$ fragments were purified by affinity chromatography on protein A (Pharmacia, Uppsala, Sweden) followed by gel filtration on Sephacryl S-200 (Pharmacia, Uppsala, Sweden). Fractions

Correspondence: D Marshall
Received 13 June 1994; revised 9 August 1994; accepted 17 August 1994

containing $F(ab')_2$ were pooled and purity was checked by SDS–PAGE.

*Biotinylation of intact and $F(ab')_2$ fragments* Caproylamido-biotin-NHS ester (Sigma, Poole, UK) in dimethylsuphoxide (DMSO) (5 mg $ml^{-1}$) was added to the antibody (1 mg $ml^{-1}$ in 0.1 M sodium bicarbonate buffer, pH 8.5) at a 24:1 molar ratio for intact A5B7 and 30:1 molar ratio for $F(ab')_2$ A5B7 and incubated at room temperature for 4 h with constant gentle agitation. The antibody was then dialysed against PBS, pH 7.4, at 4°C to remove any unreacted biotinylation reagents. This resulted in intact A5B7 with approximately ten biotins per antibody molecule and $F(ab')_2$ A5B7 with approximately eight biotins per antibody molecule, as assessed using a 4'-hydroxyazobenzene-2-carboxylic acid (HABA) dye assay (Pierce and Warriner, Chester, UK) (Green, 1965). CEA binding after biotinylation was checked by enzyme-linked immunosorbent assay (ELISA) on CEA-coated wells, and also after radiolabelling by affinity chromatography on a CEA–Sepharose column. CEA binding of radiolabelled biotinylated intact and biotinylated $F(ab')_2$ A5B7 was reduced by approximately 3% and 13% respectively in comparison with radiolabelled unmodified antibodies.

*Radiolabelling* Radiolabelling with $^{125}I$ was carried out by the Iodo-gen method (Fraker and Speck, 1978) for 20 min to a specific activity of approximately 0.5 mCi 0.5 $mg^{-1}$ protein. Chloramine T should not be used to radiolabel biotinylated antibodies as this harsh oxidation method has a detrimental effect on the biotin residues.

*Galactosylation of streptavidin*

Cyanomethyl-2,3,4,6-tetra-*O*-acetyl-1-thio-β-D-galactopyranoside (Sigma), (0.1 M in dry methanol) was mixed with 10% of 0.1 M sodium methoxide, also in dry methanol, and allowed to stand for 48 h. A 0.8 ml aliquot of this solution was added to a round-bottomed flask and evaporated to dryness, after which 5 mg streptavidin (5 mg $ml^{-1}$ in 25 mM sodium borate, pH 8.5) was added and allowed to react for 2 h. The galactosylated streptavidin was then purified by gel filtration on Sephadex G25 (Pharmacia, Uppsala, Sweden) and carbohydrate content assayed by the phenol–sulphuric acid method of Dubois *et al.* (1956) using a galactose standard. This showed the streptavidin to contain 240 μg of galactose per mg. When 5.6 ml of the galactopyranoside solution was dried and reacted with 37 mg of streptavidin (10 mg $ml^{-1}$ in 25 mM sodium borate, pH 8.5) then 71 μg galactose per mg of streptavidin resulted.

In vivo *studies*

TO nude mice bearing the LS174T xenograft, established by subcutaneous passaging of the human colon carcinoma cell line LS174T (Tom *et al.*, 1976), were injected via the tail vein with approximately 10 μg 10 $\mu Ci^{-1}$ [$^{125}I$]biotinylated antibody. Test animals were intraperitoneally injected with galactosylated streptavidin at a 10-fold molar excess of the antibody (Marshall *et al.*, 1994) 24 h after injection of biotinylated intact A5B7 or 9 h after injection of biotinylated $F(ab')_2$ A5B7. Test and control animals were bled and tissues removed to be counted for radioactivity 15 min, 1 h and 24 h after gal-streptavidin for the intact A5B7 and 15 min, 1, 15 and 24 h after gal-streptavidin for the $F(ab')_2$ A5B7.

The effect of fetuin and asialofetuin on the clearance of galactosylated streptavidin complexes was assessed in TO nude mice injected i.v. with 7 μg 7 $\mu Ci^{-1}$ [$^{125}I$]biotinylated A5B7. Twenty-four hours later test groups received either 2 mg of fetuin or 2 mg of asialofetuin i.v., followed 15 min later by galactosylated streptavidin injected i.p. at a 10-fold molar excess of the antibody. Control mice received neither fetuin nor asialofetuin. Fifteen minutes post galactosylated streptavidin injection, all mice were sacrificed and blood and tissue radioactivity assessed.

The biodistribution data were calculated as percentage injected dose per gram of tissue (% ID $g^{-1}$) and are mean values from three or four mice at each time point.

*Statistical analysis*

The non-parametric Mann–Whitney *U*-test was used to compare the difference between the groups. The results were considered significant when $P<0.05$.

### Results

*Biotinylated intact A5B7*

Control experiments show no significant difference in the biodistribution and tumour localisation of A5B7 and biotinylated A5B7 (data not shown).

We have previously shown that administration of native streptavidin will complex and clear biotinylated antibodies from the blood via the liver and spleen (Marshall *et al.*, 1994), as illustrated in Figure 1, which shows the complexes in the liver are soon degraded. The high liver radioactivity seen 1 h after streptavidin administration was reduced to below control levels by 24 h, in contrast to splenic uptake of the complexes, which 24 h after streptavidin still remained raised, resulting in a poor tumour to spleen ratio of only 3 compared with a ratio of 9.7 in the controls.

To assess whether galactosylated streptavidin (gal-streptavidin) would still complex and clear biotinylated A5B7 from the blood and to establish the route of clearance, the effect of a highly galactosylated streptavidin (240 μg galactose per mg of streptavidin), injected 24 h after the antibody was examined. Figure 2 shows a direct comparison between streptavidin and gal-streptavidin clearance of biotinylated A5B7. One hour after native streptavidin, the radioactivity level in the liver was raised from 3.2% ID $g^{-1}$ to 6.9% ID $g^{-1}$ ($P<0.04$) and splenic radioactivity increased from 2.8% ID $g^{-1}$ to 6.9% ID $g^{-1}$ compared with the control, although the increase was not statistically significant ($P<0.3$) because of a large range in levels from 1.4% ID $g^{-1}$ to 11.2% ID $g^{-1}$ for the cleared group. No high levels of splenic uptake were observed 1 h after clearance with the galactose-modified streptavidin (range 1.2–1.7% ID $g^{-1}$) and uptake of the gal-streptavidin complexes was seen in the liver only (5.6% ID $g^{-1}$). Most of the biotinylated antibody had already been

**Figure 1** Biodistribution of [$^{125}I$]biotinylated A5B7 with and without streptavidin administered 24 h after antibody injection. Test groups were dissected 1 h (▨) and 24 h (▩) after streptavidin injection (25 and 48 h post antibody injection respectively) and compared with animals without streptavidin administration at 25 (■) and 48 h (□) post antibody injection. Results are expressed as percentage injected dose per gram of tissue. Vertical bars indicate s.d.

removed from the blood 1 h after administration of the gal-streptavidin and the complexes degraded. The blood levels 1 h after clearance indicate that gal-streptavidin complexes were cleared from the blood faster than native streptavidin complexes [2.7% ID $g^{-1}$ and 5.7% ID $g^{-1}$ ($P<0.05$) respectively]. To further assess gal-streptavidin clearance of biotinylated A5B7, earlier and later time points were examined. A less galactosylated, but equally effective, streptavidin (71 μg galactose per mg of streptavidin) was used in these subsequent experiments.

Administration of gal-streptavidin 24 h after [$^{125}$I]biotinylated A5B7 produced a rapid decrease in the level of biotinylated radiolabelled antibody in the circulation. Fifteen minutes after injection of the gal-streptavidin clearing agent (Figure 3a) a 7-fold reduction in the blood radioactivity level from 16.3 to 2.3% ID $g^{-1}$ resulted ($P<0.04$), accompanied by a large rise in liver radioactivity from 5.8 to 26.7% ID $g^{-1}$ ($P<0.04$), as the galactosylated streptavidin–biotinylated antibody complexes were cleared. No significant difference in tumour radioactivity was observed at this time point. Figure 3a also shows that 1 h after administration of gal-streptavidin the radioactivity in the liver was 8.8% ID $g^{-1}$, showing the complexes to have already cleared considerably from this organ. Tumour radioactivity levels in the cleared group showed no significant difference from control animals and therefore clearance resulted in a much improved tumour to blood ratio from 1.9 to 7.4 ($P<0.04$) at only 25 h after injection of the radiolabelled antibody.

Figure 3b shows that 24 h after injection of the gal-streptavidin the blood and all normal tissues had significantly lowered ($P<0.05$) levels of radioactivity. Although the level of radioactivity in the tumour had also fallen, this was to a lesser extent than in the blood, and thus a much improved ($P<0.05$) tumour to blood ratio of 13.2 was achieved compared with only 2.8 without clearance, as shown in Figure 4. The large error bar was due to a wide range of tumour to blood ratios being observed after gal-streptavidin clearance, the worst being 4.7, while the best tumour to blood ratio was 19.9.

*Biotinylated A5B7 F(ab')$_2$*

Control experiments show no significant difference in the biodistribution and tumour localisation of A5B7 F(ab')$_2$ and biotinylated A5B7 (data not shown).

**Figure 2** Biodistribution of [$^{125}$I]biotinylated A5B7 1 h post clearing with streptavidin ( ▨ ) or gal-streptavidin ( □ ). Test groups were injected with the clearing agent 24 h after antibody injection and compared with animals that did not receive clearing agent ( ■ ). Results are expressed as percentage injected dose per gram of tissue. Vertical bars indicate s.d.

Figure 5 shows that the early clearance pattern of [$^{125}$I]-biotinylated F(ab')$_2$ after gal-streptavidin was similar to the pattern seen with [$^{125}$I]biotinylated intact A5B7, with the complexes being quickly taken up and rapidly cleared by the liver (10% ID $g^{-1}$ and 5.3% ID $g^{-1}$ was seen in the liver

**Figure 3** Biodistribution of [$^{125}$I]biotinylated A5B7 **a**, 15 min ( ▨ ) and 1 h ( □ ) post gal-streptavidin administration and **b**, 24 h ( ▨ ) post gal-streptavidin administration. Test groups were injected with gal-streptavidin 24 h after antibody injection and compared with animals that did not receive gal-streptavidin ( ■ ). Results are expressed as percentage injected dose per gram of tissue. Vertical bars indicate s.d.

**Figure 4** Tissue to blood ratios of [$^{125}$I]biotinylated A5B7 24 h post gal-streptavidin administration. Test group ( ▨ ) was injected with gal-streptavidin 24 h after antibody injection and compared with animals that did not receive gal-streptavidin ( ■ ). Vertical bars indicate s.d.

**Figure 5** Biodistribution of [$^{125}$I]biotinylated A5B7 F(ab′)$_2$ 15 min (▨) and 1 h (□) post gal-streptavidin administration. Test groups were injected with gal-streptavidin 9 h after antibody injection and compared with animals that did not receive gal-streptavidin (■). Results are expressed as percentage injected dose per gram of tissue. Vertical bars indicate s.d.

**Figure 6** Biodistribution of [$^{125}$I]biotinylated A5B7 F(ab′)$_2$ (**a**) 15 h and (**b**) 24 h post gal-streptavidin administration. The test group (▨) was injected with gal-streptavidin 9 h after antibody injection and compared with animals that did not receive gal-streptavidin (■). Results are expressed as percentage injected dose per gram of tissue. Vertical bars indicate s.d.

15 min and 1 h after gal-streptavidin, compared with 3.8% ID g$^{-1}$ in the controls). Levels of biotinylated F(ab′)$_2$ in the blood were lowered considerably ($P$<0.03) to 4.4% ID g$^{-1}$ and 3.4% ID g$^{-1}$ at 15 min and 1 h respectively in comparison with 11.7% ID g$^{-1}$ without clearance.

Biodistribution of [$^{125}$I]biotinylated F(ab′)$_2$ at later time points is shown in Figure 6, using a different batch of F(ab′)$_2$ which gave superior tumour localisation to the previous experiments. Fifteen hours after injection of gal-streptavidin (24 h after biotinylated F(ab′)$_2$ injection) all normal tissues had significantly lowered ($P$<0.03) levels of radioactivity, with blood levels reduced from 5.6% ID g$^{-1}$ to 0.26% ID g$^{-1}$ (21.5-fold reduction), as shown in Figure 6a. Tumour levels of radioantibody were reduced non-significantly ($P$<0.2), from 19.8% ID g$^{-1}$ to 11.9% ID g$^{-1}$, and therefore a 12.9-fold increase ($P$<0.03) in the tumour to blood ratio resulted. Figure 6b shows the biodistribution of the [$^{125}$I]biotinylated F(ab′)$_2$ 24 h after gal-streptavidin administration. The tumour was the only tissue with high levels of radioactivity and, although this level was 2-fold lower ($P$<0.03) than in controls without the gal-streptavidin clearing agent, the large reduction in blood radioactivity levels means that the tumour to blood ratio was greatly improved from 4.9 to 33.2 ($P$<0.03), as shown in Figure 7.

### *Effect of fetuin and asialofetuin*

Table I shows that asialofetuin, which is known to bind to the asialoglycoprotein receptor (Ashwell and Morell, 1974), inhibits the removal of gal-streptavidin-complexed [$^{125}$I]biotinylated A5B7 from the blood. [$^{125}$I]biotinylated A5B7 is shown to have cleared very rapidly via the liver 15 min after injection of gal-streptavidin (control group), whereas 2 mg of asialofetuin injected 15 min prior to administration of gal-streptavidin inhibited blood clearance of the gal-streptavidin–biotinylated A5B7 complexes [8.9% ID g$^{-1}$ remained in the blood compared with only 1.9% ID g$^{-1}$ ($P$<0.03) in

**Figure 7** Tissue to blood ratios [$^{125}$I]biotinylated A5B7 F(ab′)$_2$ 24 h post gal-streptavidin administration. The test group (▨) was injected with gal-streptavidin 9 h after antibody injection and compared with animals that did not receive gal-streptavidin (■). Vertical bars indicate s.d.

**Table I** The effect of an asialoglycoprotein receptor inhibitor on the percentage injected dose per gram (% ID g$^{-1}$) of [$^{125}$I]biotinylated A5B7 in the blood and liver after clearance with galactosylated streptavidin

| | *Blood % ID g$^{-1}$* | *Liver % ID g$^{-1}$* |
|---|---|---|
| Control | 1.9 ± 1.2 | 25.9 ± 5.3 |
| Asialofetuin | 8.9 ± 1.2 | 9.6 ± 3.8 |
| Fetuin | 2.2 ± 1.8 | 25.5 ± 4.4 |

the control group]. The very high liver uptake usually seen with complex clearance was also reduced after asialofetuin injection from 25.9 to 9.6% ID $g^{-1}$ ($P<0.03$). Fetuin (2 mg) injected 15 min prior to gal-streptavidin administration had no effect on blood clearance or liver uptake of the complexes.

### Discussion

Our previous work focused on how the degree of biotinylation affects the biodistribution of [$^{125}$I]biotinylated antibody after administration of streptavidin to clear circulating radiolabelled antibody (Marshall *et al.*, 1994). A large improvement in the tumour to blood ratio was achieved when nine or more biotins were conjugated to the antibody (four biotin residues resulted in no improvement in the tumour to blood ratio). The major problem noted was the high and persistent levels of radioactivity in the spleen, and a method to circumvent this splenic uptake was required.

Galactose conjugated to antibodies has been shown to greatly increase their clearance from the circulation via the liver (Mattes, 1987; Sharma *et al.*, 1990). Mannose residues have been successfully conjugated onto streptavidin, without impairing biotin binding, to divert biotinylated oligodeoxynucleotides to macrophages (Bonfils *et al.*, 1992). In this work we have conjugated galactose residues onto streptavidin with a view to directing streptavidin complexes to the asialoglycoprotein receptor of hepatocytes. Figure 2 shows that the formation of streptavidin–biotinylated antibody complexes was not impaired by conjugation of galactose to streptavidin, giving good blood clearance, and we successfully abolished the high splenic uptake of complexes previously seen with macrophage-dependent clearance using native streptavidin (Marshall *et al.*, 1994). The asialoglycoprotein receptor binds galactosylated proteins rapidly and strongly ($K_a$ is of the order of $10^9$ $M^{-1}$, the exact value being dependent upon the ligand) and are very abundant (100 000–500 000 receptors per cell) (Schwartz, 1984), and therefore this specific receptor-mediated endocytosis may account for the more rapid removal of gal-streptavidin complexes from the blood than uptake due to phagocytosis by macrophages when native streptavidin was used. The minimal involvement of non-target organs in clearing the radioantibodies and the rapidity with which large tumour to blood ratios are achieved with [$^{125}$I]biotinylated A5B7 makes this scheme ideal for imaging. $^{131}$I-labelled antibodies are expected to show similar results and Figure 4 illustrates the great improvement in the potential therapeutic ratio which can be achieved 48 h after antibody injection when gal-streptavidin clearance is used. Iodine-labelled antibodies are known to be deiodinated *in vivo* and clear rapidly from the liver. Antibodies labelled with other therapeutic isotopes, such as radiometals, may not be cleared in the same manner and therefore the effect of gal-streptavidin clearance on radiometal-labelled antibodies also needs to be assessed.

This clearance mechanism is of great interest because of its potential universal application to any anti-tumour antibody (lysine residues are readily available for biotinylation), and it was also of interest to establish the effect of clearance on the biodistribution and tumour localisation of a $F(ab')_2$ fragment. The clearing agent was given when tumour localisation of the antibody was at a peak, which is at an earlier time point for $F(ab')_2$ fragments than the peak seen with whole IgG (Pedley *et al.*, 1993), and therefore allowed earlier administration of gal-streptavidin when using $F(ab')_2$ fragments. Thus the high and potentially damaging dose of radioactivity received during the time prior to administration of the clearing agent would be for a much shorter time if $F(ab')_2$ fragments were used.

The early clearance pattern of biotinylated $F(ab')_2$ with gal-streptavidin was similar to that seen with intact antibody (transient high liver activity as the complexes are rapidly cleared). Later time points revealed that 24 h after gal-streptavidin clearance a smaller proportion of biotinylated $F(ab')_2$ was removed from the tumour than the proportion of intact A5B7 removed. Figure 3b shows that localisation of the biotinylated A5B7 in the tumour was reduced to only 37% of that seen without clearance, compared with a loss of 50% of the biotinylated $F(ab')_2$ from the tumour 24 h after gal-streptavidin (Figure 6b). This better retention of biotinylated $F(ab')_2$ in the tumour, together with lower blood levels, resulted in a much improved tumour to blood ratio of 33:1 when compared with the ratio obtained 24 h after clearance of biotinylated intact antibody (13.2:1), indicating that $F(ab')_2$ with clearance is superior to the cleared intact antibody and therefore preferable for imaging studies. For radioimmunotherapy a high dose of antibody at the tumour, as well as a large tumour to blood ratio, is required. A comparison of the therapeutic efficacy of intact *vs* $F(ab')_2$ A5B7 has shown that the fragments, with higher tumour to blood ratios, gave twice the radiation dose to the tumour than was delivered by the intact antibody, for a similar radioactivity dose to the blood and hence the bone marrow (Pedley *et al.*, 1993). A comparative dosimetry study of biotinylated intact and $F(ab')_2$ A5B7 with and without clearance is required in order to select the optimal therapeutic modality.

The size of an anti-tumour antibody is known to affect its biodistribution and clearance (Harwood *et al.*, 1985; Yokota *et al.*, 1993) and the absolute amount of antibody which localises to the tumour has been shown in this work and by others (Pedley *et al.*, 1993; Vogel *et al.*, 1993) to be less for a fragment than an intact antibody. Pervez *et al.* (1988) and Yokota *et al.* (1992) have carried out autoradiographic studies to examine how penetration into the tumour differs with the size of the antibody. These studies imply that at early time points after antibody injection (comparable with time of administration of gal-streptavidin in these experiments), $F(ab')_2$ will have penetrated deeper into the tumour than whole IgG. The exact mechanism for the reduction in antibody localisation in the tumour after administration of a clearing agent is unknown, but could be circulatory clearance combined with gal-streptavidin gaining access to the tumour, where it can complex with tumour-bound biotinylated antibody, causing dissociation from the tumour and clearance from the body. Deeper penetration of $F(ab')_2$ would mean that antibody fragments are further from blood vessels than whole IgG and therefore less likely to be available for complexation with gal-streptavidin, resulting in a greater proportion of the $F(ab')_2$ remaining in the tumour after clearance.

Evidence that the asialoglycoprotein receptor on hepatocytes is involved in the clearance mechanism is shown in Table I. Injection of asialofetuin 15 min prior to injection of the gal-streptavidin was seen to inhibit blood clearance, together with inhibiting liver uptake, of the gal-streptavidin–biotinylated A5B7 complexes. Injection of fetuin had no such inhibiting effect, thus indicating that the asialoglycoprotein receptor of liver hepatocytes, known to bind asialofetuin but not fetuin (Ashwell and Morell, 1974), is involved in clearing the complexes.

This study shows that the properties of not only the antibody, but also the associated proteins, direct the clearance mechanism. Gal-streptavidin directed clearance via the hepatocytes, whereas immune complexes of biotinylated antibodies are normally cleared via the RES. Rifai *et al.* (1982) also demonstrated that immune complexes could be directed to either the parenchymal or non-parenchymal cells of the liver depending on whether the antigen was rich in galactose or mannose. Work by Krantz *et al.* (1976) showed a direct relationship between the amount of galactose conjugated to a protein and its binding to liver membranes. In our work, a decrease in the degree of galactosylation of the streptavidin from 240 to 71 μg of galactose per mg of streptavidin did not effect the clearing pattern of the gal-streptavidin–biotinylated antibody complexes, although it is possible that any further reduction in the degree of galactosylation of streptavidin could reduce its effectiveness in clearing biotinylated compounds, and further work to establish the minimum degree of galactose conjugation required for effective clearance has yet to be carried out.

Galactosylation could be beneficial for other avidin–biotin tumour targeting schemes. Ogihara-Umeda *et al.* (1993, 1994) used avidin to clear biotin-bearing liposomes. As with streptavidin clearance of biotinylated antibodies, high splenic uptake was seen after clearance and therefore galactosylated streptavidin should circumvent this problem. Streptavidin has also been used in two-step pretargeting in which radiolabelled streptavidin is injected after preadministration of cold biotinylated anti-tumour antibody. Saga *et al.* (1994) reported liver and splenic radioactivity levels to be higher after pretargeting than when radiolabelled streptavidin alone was injected as a result of the formation of streptavidin–biotinylated antibody complexes and their uptake by the RES. Galactosylation of the streptavidin would limit this uptake to the liver alone. Gal-streptavidin may also be of use in the three-step protocol of Paganelli *et al.* (1990*b*, 1991), in which avidin is used to clear cold biotinylated antibody before administration of the isotope.

We have previously shown that the administration of streptavidin will rapidly clear biotinylated radioantibodies from the circulation via the liver and spleen, producing much improved tumour to blood ratios compared with antibody alone. The liver rapidly dehalogenates the iodinated complexes and the free iodine is excreted, whereas complexes cleared via the spleen tend to be metabolised at a much slower rate. By using a galactosylated form of streptavidin, we have enhanced the antibody clearance by removing accumulation of radioactive antibody complexes which persist in the spleen without increasing liver accumulation (Figure 2 shows that liver radioactivity is approximately the same 1 h after both streptavidin and gal-streptavidin clearance). Galactosylation of the clearing agent, and thus exploitation of the asialoglycoprotein receptors on hepatocytes of the liver, reduces damage which may be caused by prolonged exposure of radioactivity to normal tissues. Biotinylation, via lysine residues, is a mild procedure (only limited reduction in the immunoreactivity of A5B7 was found) and should be applicable to any anti-tumour antibody. This universal clearance scheme, with increased tumour to blood ratios and minimal involvement of normal tissues, should greatly improve antibody-targeted imaging and therapy of cancer. It is of use not only for radioimmunotherapy but also for targeted therapy of toxins/drugs including antibody-directed enzyme pro-drug therapy (ADEPT) (Bagshawe, 1987), when very low levels of the antibody enzyme conjugate in the blood and normal tissues are essential before the prodrug can be administered. There is also potential for controlling clearance of any pharmaceutical drug which can be biotinylated. This would be useful for controlling the duration of exposure to a drug or for clearing drugs via the liver when their normal organ of clearance (e.g. the kidney) is impaired.

**Acknowledgements**

A5B7 was kindly provided by Celltech Limited, Slough, UK. This work was supported by the Cancer Research Campaign.

**References**

ASHWELL G AND MORELL AG. (1974). The role of surface carbohydrates in the hepatic recognition and transport of circulating glycoproteins. *Adv. Enzymol.*, **41**, 99–128.

BAGSHAWE KD. (1987). Antibody directed enzymes revive anticancer prodrugs concept. *Br. J. Cancer*, **56**, 531–532.

BENACERRAF B, SEBESTYEN M AND COOPER NS. (1959). The clearance of antigen antibody complexes from the blood by the reticulo-endothelial system. *J. Immunol.*, **82**, 131–137.

BEGENT RHJ, KEEP PA, GREEN AJ, SEARLE F, BAGSHAWE KD, JEWKES RF, JONES BE, BARRATT GM AND RYMAN BE. (1982). Liposomally entrapped second antibody improves tumour imaging with radiolabelled (first) antitumour antibody. *Lancet*, **ii**, 739–742.

BONFILS E, MENDES C, ROCHE A-C, MONSIGNY M AND MIDOUX P. (1992). Uptake by macrophages of a biotinylated oligo-α-deoxythymidylate by using mannosylated streptavidin. *Bioconj. Chem.*, **3**, 277–284.

DUBOIS M, GILLES KA, HAMILTON JK, REBERS PA AND SMITH F. (1956). Colorimetric method for determination of sugars and related substances. *Anal. Chem.*, **28**, 350–356.

FRAKER PJ AND SPECK JC. (1978). Protein and cell membrane iodinations with a sparingly soluble chloroamide, 1,3,4,6-tetrachloro-3a,6-diphenylglycoluril. *Biochem. Biophys. Res. Commun.*, **80** (4), 849–857.

GREEN NM. (1965). A spectrophotometric assay for avidin and biotin based on binding of dyes by avidin. *Bichem. J.*, **94**, 23c–24c.

HARWOOD PJ, BODEN J, PEDLEY RB, RAWLINS G, ROGERS GT AND BAGSHAWE KD. (1985). Comparative tumour localization of antibody fragments and intact IgG in nude mice bearing a CEA-producing human colon tumour xenograft. *Eur. J. Cancer Clin. Oncol.*, **21**, 1515–1522.

KRANTZ MJ, HOLTZMAN NA, STOWELL CP AND LEE YC. (1976). Attachment of thioglycosides to proteins: enhancement of liver membrane binding. *Biochemistry*, **15**, 3963–3968.

MARSHALL D, PEDLEY RB, BODEN JA, BODEN R AND BEGENT RHJ. (1994). Clearance of circulating radio-antibodies using streptavidin or second antibodies in a xenograft model. *Br. J. Cancer*, **69**, 502–507.

MATTES JM. (1987). Biodistribution of antibodies after intraperitoneal or intravenous injection and effect of carbohydrate modifications. *J. Natl Cancer Inst.*, **79**, 855–863.

OGIHARA-UMEDA I, SASAKI T AND NISHIGORI H. (1993). Active removal of radioactivity in the blood circulation using biotin-bearing liposomes and avidin for rapid tumour imaging. *Eur. J. Nucl. Med.*, **29**, 170–172.

OGIHARA-UMEDA I, SASAKI T, TOYAMA H, ODA K, SENDA M. AND NISHIGORI H. (1994). Rapid tumor imaging by activie background reduction using biotin-bearing liposomes and avidin. *Cancer Res.*, **54**, 463–467.

PAGANELLI G, STELLA M, DE NARDI P, MAGNANI P, ZITO F, SICCARDI G, DI CARLO V AND FAZIO F. (1990*a*). A new method for faster blood clearance in radioimmuno-guided surgery. *J. Nucl. Med. Allied. Sci.*, **35**, 88–89.

PAGANELLI G, PERVEZ S, SICCARDI G, ROWLINSON G, DELEIDE G, CHIOLERIO F, MALCOVATI M, SCASSELLATI GG AND EPENETOS AA. (1990*b*). Intraperitoneal radio-localisation of tumours pre-targeted by biotinylated monoclonal antibodies. *Int. J. Cancer*, **45**, 1184–1189.

PAGANELLI G, MAGNANI P, ZITO F, VILLA E, SUDATI F, LOPALCO L, ROSSETI L, MALCOVATI M, CHIOLERIO F, SECCAMANI E, SICCARDI AG AND FAZIO F. (1991). Three-step monoclonal antibody tumor targeting in carcinoembryonic antigen-positive patients. *Cancer Res.*, **51**, 5960–5966.

PEDLEY RB, BODEN J, KEEP PA, HARWOOD PJ, GREEN AJ AND ROGERS GT. (1987). Relationship between tumour size and uptake of radiolabelled anti-CEA in a colonic tumour xenograft. *Eur. J. Nucl. Med.*, **13**, 197–202.

PEDLEY RB, DALE R, BODEN JA, BEGENT RHJ, KEEP PA AND GREEN AJ. (1989). The effect of second antibody clearance on the distribution and dosimetry of radiolabelled anti-CEA antibody in a human colonic tumour xenograft model. *Int. J. Cancer*, **43**, 713–718.

PEDLEY, RB, BODEN JA, BODEN R, DALE R AND BEGENT RHJ. (1993). Comparative radioimmunotherapy using intact or $F(ab')_2$ fragments of $^{131}I$ anti-CEA antibody in a colonic xenograft model. *Br. J. Cancer*, **68**, 69–73.

PERVEZ, S, EPENETOS AA, MOOI WJ, EVANS DJ, ROWLINSON G, DHOKIA B AND KRAUSZ T. (1988). Localization of monoclonal antibody AUA1 and its $F(ab')_2$ fragments in human tumour xenografts: an autoradiographic and immunohistochemical study. *Int. J. Cancer*, Suppl. 3, 23–29.

RIFAI A, FINBLOOM DS, MAGILAVY DB AND PLOTZ PH. (1982). Modulation of the circulation and hepatic uptake of immune complexes by carbohydrate recognition systems. *J. Immunol.*, **128**, 2269–2275.

ROGERS JC AND KORNFELD S. (1971). Hepatic uptake of proteins coupled to fetuin glycopeptide. *Biochem. Biophys. Res. Commun.*, **45**, 622–629.

SAGA T, WEINSTEIN JN, JEONG JM, HEYA T, LEE JT, LE N, PAIK CH, SUNG C AND NEUMANN RD. (1994). Two-step pretargeting of experimental lung metastases with biotinylated antibody and radiolabeled streptavidin. *Cancer Res.*, **54**, 2160–2165.

SCHWARTZ AL. (1984). The hepatic asialoglycoprotein receptor. *CRC Crit. Rev. Biochem.*, **16**, 207–233.

SHARKEY RM, PRIMUS FJ AND GOLDENBERG DM. (1984). Second antibody clearance of radiolabeled antibody in cancer radioimmunodetection. *Proc. Natl Acad. Sci. USA*, **81**, 2843–2846.

SHARMA, SK, BAGSAHWE KD, BURKE PJ, BODEN RW AND ROGERS, GT. (1990). Inactivation and clearance of an anti-CEA carboxypeptidase G2 conjugate in blood after localisation in a xenograft model. *Br. J. Cancer*, **61**, 659–662.

SINITSYN VV, MAMONTOVA AG, CHECKNEVA YY, SHNYRA AA AND DOMOGATSKY SP. (1989). Rapid blood clearance of biotinylated IgG after infusion of avidin. *J. Nucl. Med.*, **30**, 66–69.

THORNBERG RW, DAY JF, BAYNES JW AND THORPE SR. (1980). Carbohydrate-mediated clearance of immune complexes from the circulation. *J. Biol. Chem.*, **255**, 6820–6825.

TOM BH, RUTZKY LP, JAKSTYS MM, OYASU R, KAYE CI AND KAHAN BD. (1976). Human colonic adenocarcinoma cells. *In Vitro*, **12** (3), 180–191.

VOGEL C-A, BISCHOF-DELALOYE A, MACH J-P, PELEGRIN A, HARDMAN N, DELALOYE B AND BUCHEGGAR F. (1993). Direct comparison of a radioiodinated intact chimeric anti-CEA MAb with its $F(ab')_2$ fragment in nude mice bearing different human colon cancer xenografts. *Br. J. Cancer*, **68**, 684–690.

YOKOTA T, MILENIC DE, WHITLOW M AND SCHLOM J. (1992). Rapid tumor penetration of a single-chain Fv and comparison with other immunoglobulin forms. *Cancer Res.*, **52**, 3402–3408.

YOKOTA T, MILENIC DE, WHITLOW M, WOOD JF, HUBERT SL AND SCHLOM J. (1993). Microautoradiographic analysis of the normal organ distribution of radioiodinated single-chain Fv and other immunoglobulin forms. *Cancer Res.*, **53**, 3776–3783.